# Global Potential Distribution of Invasive Species *Pseudococcus viburni* (Hemiptera: Pseudococcidae) under Climate Change

**DOI:** 10.3390/insects15030195

**Published:** 2024-03-14

**Authors:** Jiufeng Wei, Minmin Niu, Hanxi Zhang, Bo Cai, Wei Ji

**Affiliations:** 1 College of Plant Protection, Shanxi Agricultural University, Jinzhong 030801, China; wjfeng@nwsuaf.edu.cn (J.W.); niuminmin@yeah.net (M.N.); 2 College of Horticulture and Landscape Architecture, Tianjin Agricultural University, Tianjin 300300, China; zhanghanxi_0929@163.com; 3 Post-Entry Quarantine Station for Tropical Plant, Haikou Customs District, Haikou 570311, China; caibohnciq@163.com; 4 College of Horticulture, Shanxi Agricultural University, Taigu 030801, China

**Keywords:** MaxEnt, potential distribution model, worldwide, environmental variables, alien species

## Abstract

**Simple Summary:**

*Pseudococcus viburni* (Signoret) is an important invasive species that is recorded in more than 60 countries worldwide and feeds on nearly 91 families and 259 genera. However, the potential distribution range and management strategies for this pest are still poorly understood. Based on historical distribution data and environmental factors, the present study obtained the potentially suitable areas of *P. viburni* under different climate change scenarios by MaxEnt. The total area of suitable habitat areas will also show an increased trend in future climatic conditions. In order to control the spread of *P. viburni*, we need to strengthen the monitoring and quarantine measures at the Southern ports.

**Abstract:**

The potential distribution range and management strategies for *P. viburni* are poorly understood. Based on historical distribution data and environmental factors, the present study predicted the potentially suitable areas for *P. viburni* spread under different climate change scenarios using MaxEnt (maximum entropy). The results showed that precipitation of the coldest quarter (Bio19), precipitation seasonality (Bio15), and mean temperature of the wettest quarter (Bio8) were the most important environmental factors determining the distribution of *P. viburni*. Under the current climate conditions, its potential suitable areas are southern China, the whole of Japan, North America (especially the eastern part of the United States), the southwestern part of South America, the Mediterranean coast and most of Europe, the central part of Africa, i.e., the south of the Sahara Desert, and most of the southern coast of Australia. The total area of habitats suitable for this insect pest is predicted to be increased in the future. In order to prevent *P. viburni* transmission and spread, there is a need to strengthen the monitoring and quarantine measures against this pest at the Southern ports.

## 1. Introduction

Mealybugs (Hemiptera: Pseudococcidae) are important plant pests worldwide [1]. They are small sap-sucking insects, reproduce quickly, and have a large population [2]. Once harmed, it will lead to leaf yellowing, defoliation, reduced plant growth, and even the entire plant withering and dying. Indirectly, they can also damage plants as insect-vectors of plant disease, such as: grapevine leafroll-associated viruses (GLRaVs) [3,4] and cacao mild mosaic virus [5]. In addition, the honeydew secreted by scale insects contaminates host plants and breeds sooty mold, seriously reducing the yield and quality of fruit and affecting the greening effect of street trees and flowers. On the other hand, mealybugs are small and live in seclusion, which can easily spread to new areas by the transporting of seedlings, leading to outbreaks and disasters. Therefore, many mealybugs are regarded as quarantine objects, and quarantine measures are strictly implemented to prevent their transmission and spread [6].

*Pseudococcus viburni* (Signoret, 1875) is a worldwide species, found in 60 countries [7], also commonly known as the obscure mealybug, previously recorded in Brazil only in the states of Minas Gerais, Rio de Janeiro, and São Paulo [8,9,10]. It is possible that *P. viburni* was brought into Europe on potatoes from South America via the Canary Islands in the sixteenth century [11]. The mealybug was first described from France, as *Dactylopius viburni* Signoret, 1875. This species was spread from its area of origin early, by human transport of infested plants; subsequently, this has made it difficult to work out its area of origin. *P. viburni* has a very wide host range, nearly 91 plant families and 259 genera [7], including important economic crops such as apples, citrus, grapes, and tomatoes, as well as papaya and potatoes [12].

Climate is considered to be the main factor affecting species’ large-scale distribution [13,14]. Predicting species’ geographical distribution and their response to climate change is significant for biodiversity conservation and sustainable ecosystems development [15]. The potential impact of climate change on the predicted range and distribution of invasive species is well recognized; research efforts focus mainly on the spatial characteristics of species distribution, in order to facilitate management and protection [16,17].

MaxEnt (maximum entropy) is a machine learning model used to find the probability of maximum entropy distribution, which can be used to predict the potential distribution of target species that meet the maximum entropy under various conditions [18]. This software estimated the potential distribution of focus species by environmental variables and a small amount of species distribution data. Specifically, MaxEnt predicts the potential distribution by analyzing the location data of the target species (a dependent variable) as a function of environmental variables. Distribution data are generally occurrence data obtained through field investigation or literature and network, and the existing information such as land cover, forest type, ecological zone, distance, geographical characteristics, and climate data can be used as the source of environmental variables [19].

In this current study, the MaxEnt software was employed to predict the world’s potential geographical distribution range of *P. viburni* under different climate change scenarios. The main objectives were:

(1) to identify the mainly environmental factors affecting the potential distribution of *P. viburni*;
(2) to observe the trends of suitable habitat range under climate change background;
(3) to provide a theoretical reference for the relevant departments to formulate corresponding quarantine measures and ensure agriculture safety.

## 2. Materials and Methods

### 2.1. Data on Occurrence of the Species

**Figure 1:**
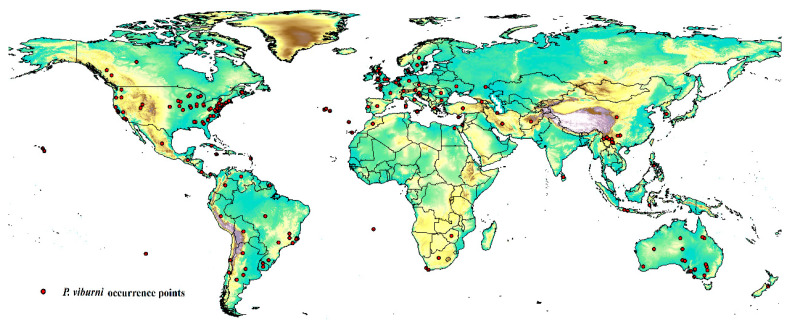
Geographical occurrence points of *Pseudococcus viburni*.

The occurrence data of *P. viburni* were primarily obtained from two sources: the Global Biological Diversity Information Facility (GBIF: https://www.gbif.org/ (accessed on 10 November 2023)) (doi:10.15468/dl.yp7ene) [20] and Scalenet [7]. The Scalenet is a literature-based model of scale insect biology and systematics, which included the geographic distribution information for nearly all scale insects all over the world. Thus, the initial data included 216 locations (Table S1). To reduce the impact of bias on the MaxEnt software, the R package “DISMO” was used to screen and delete distribution points with duplicate occurrences and potential errors [21]. The “Fishnet” method was used to create a small grid with a side length of 5 km to examine the remaining distribution points, and then randomly select location points in the grid with multiple distribution points. This work was conducted in ArcGIS 10.2 (ESRI, Redlands, CA, USA). After filtering the data, 202 location points were obtained for the final analysis, as shown in Figure 1 and Table S2.

### 2.2. Selection and Comparison of Environmental variables

The environmental variables used in this study were climate variables obtained from the World Environment Variable database (WorldClim 1.4; http://www.worldclim.org (accessed on 15 November 2023)) [22]. The data included 19 bioclimatic variables (Bio1-Bio19) (Table 1) of the current and future scenarios with a spatial resolution was 2.5 arc minute (~5 km). For future climatic conditions, we used four different representative concentration pathways (RCPs), RCP2.6, RCP4.5, RCP6.0, and RCP8.5 of two future periods, i.e., 2050s and 2070s. Different concentration pathways represent different radiative forcings and different CO_2_ equivalent concentrations that affect the life and physiological activities of insects. Three global climate models (GCMs) were selected for each representative concentration pathway, including HADGEM2-AO, BCC-CSM1-1, and MIROC5, to assess their future potential distribution.

There is usually multicollinearity among multiple variables, which reduces the transferability of the model [23]. In order to minimize the effects of highly correlated variables, Pearson correlation analysis was used for each pairwise comparison of all selected environmental variables for removal of some of the redundant and less important variables. Highly correlated variables (r^2^ ≥ |0.8|) for *P. viburni* were removed from original environmental variables, which filtered those below the threshold values for final analysis (Table S3). The Pearson correlation analysis was implemented by SPSS statistics 19.0. Finally, Bio2, Bio8, Bio15, Bio18, and Bio19 were retained for further analysis.

### 2.3. Model Construction

The MaxEnt software (version 3.4.1) was used in this study to map the potential distribution of *P. viburni* [15]. Generally, the default settings of MaxEnt software generate overfitting models [24]. Therefore, Feature Class (FC) and Regular Multiplier (RM) were used to optimize the model. The characteristic type represents the different conversions of the coordinated variables [25], including linear (L), multiplication (P), hinge (H), threshold (T), and quadratic (Q) [26]. RM can reduce the model overfitting. We used the R package “ENMeval” [27] to test whether the parameters were overfitted and selected the combination of multiplication and elements based on these results [28]. The RM value range was 0.5 to 4.0 in increments of 0.5, and the FC had eight different combinations (L, LQ, LQP, QHP, LQH, LQHP, QHPT, LQHPT). The “checkerboard2” approach was applied by calculating the standardized Akaike information criterion coefficient (AICc), and the lowest delta AICc scores were selected to run the final MaxEnt models [29].

**Figure 2:**
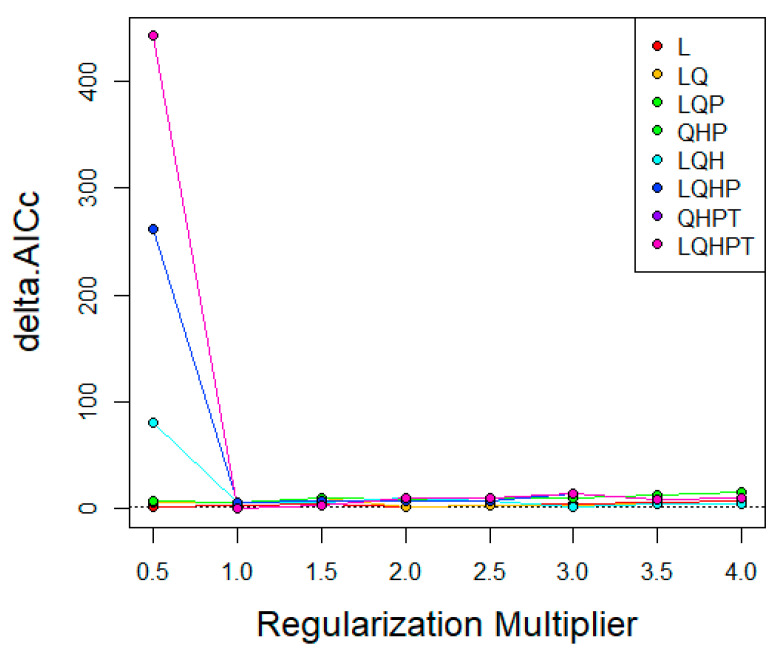
AICc value of parameter combination (FC) calculated based on ENMeval.

In the research of *P. viburni*, the best parameters of the FC and RM were set to QPHT and 1, respectively (Figure 2, Table S4). In addition, the logistic output of MaxEnt was used in current study. The suitable and unsuitable habitats or binary presence/absence maps for *P. viburni* were defined by a 10th percentile training presence logistic threshold. This threshold has been widely applied in species distribution modeling, especially when data were collected by different collectors [30]. The jackknife test was used to assess the effects of each environmental variable. The number of background points was set to be 10,000 and the model was repeated 10 times to take the average, and the rest were set by default. A 10-fold cross-validation was used to run MaxEnt to prevent random errors [31] based on species occurrence data and environmental variables. This study employed the same threshold to define the suitable and unsuitable habitats for this species. For predicting future habitat areas, the average values obtained from the three global climate models (GCMs) were used to construct the distribution map of habitat areas.

### 2.4. Identification of Potential Habitat Threshold

Based on a logistic output format, MaxEnt produced a worldwide prediction map, which provided a continuous habitat suitability index ranging from 0 (unsuitable) to 1 (highly suitable). A 10th percentile training presence logistic threshold (0.3062) was used to perform suitability level (defining suitable and unsuitable areas) classification on the predicted results [32]. Suitable habitat areas were divided into four levels: (1) unsuitable habitats where suitability area was below threshold; (2) threshold—0.4, which represented low habitat suitability; (3) 0.4–0.6 threshold represented moderate habitat suitability area; and (4) 0.6–1 threshold represented high habitat suitability area.

### 2.5. Model Evaluation

Several indicators were applied to evaluate MaxEnt models including the receiver operating characteristic (AUC), kappa statistic (Kappa), the true skill statistic (TSS), and the partial AUC (pAUC) [33]. Among them, AUC is the most widely used one. However, there are disadvantages to this approach due to equal weighting of omission and commission errors, and even AUC cannot provide information on the spatial distribution of model errors. [29,34]. Therefore, in this study, the partial receiver operating characteristic (pROC) metric approach was chosen to evaluate the performance of the model. The Niche toolbox (http://shiny.conabio.gob.mx:3838/nichetoolb2/ (accessed on 12 December 2023)) was used for this processing, and parameters were set up to be assessed with 1000 repetitions and E = 0.05 [35].

## 3. Results

### 3.1. Model Performance Evaluation

This work predicted the habitats suitable for *P. viburni* using species distribution modeling based on the known occurrence points of the species and climatic factors (including current and different model climatic variables). The results of the performance of the model have been proved by pROC tests to have more accurate prediction potential (Figure S1). The mean value for partial AUC at 0.05 was 0.74998 for *P. viburni* (*p* < 0.001).

### 3.2. Climate Variables

The model results showed that the precipitation index was the most important factor affecting the geographical distribution of *P. viburni* (Table 2). Further, it showed that the Precipitation of Coldest Quarter (Bio19) contributed the most to the model, accounting for about 70.7%; precipitation seasonality (Bio15) and mean temperature of the wettest quarter (Bio8) contributed 12.6% and 10.8% to the model, respectively.

### 3.3. Response Curves

**Figure 3:**
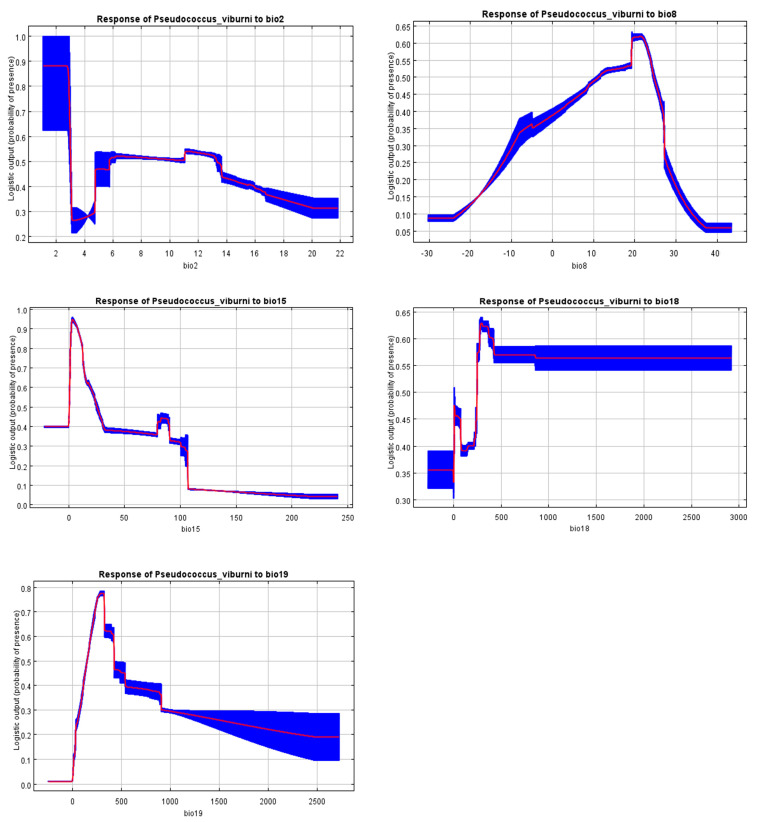
Response curves reflecting the relationship between the potential distribution of *Pseudococcus viburni* and environmental variables. The curves show the mean response of the 10 replicate MaxEnt runs (red) and the mean ± SD (blue).

The response curves indicated how the climatic suitability of *P. viburni* was changed with the five selected environmental variables (Figure 3). Based on these response curves, climate variables associated with habitat suitability areas were 1–2.5 °C for Bio2, 19–24 °C for Bio8, 0–24 for Bio15, 250–400 mm for Bio18, and 200–400 mm for Bio19.

### 3.4. Current Suitable Area

**Figure 4:**
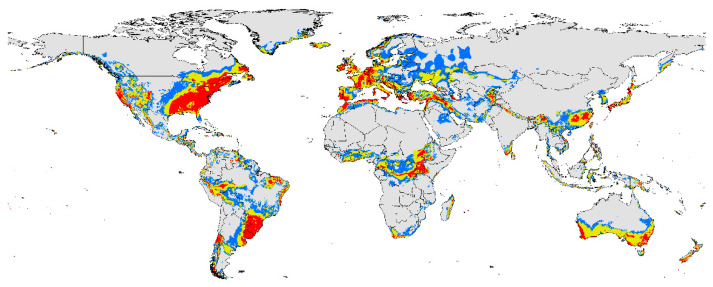
The habitat suitability areas of *Pseudococcus viburni* under current climatic conditions. Gray, unsuitable habitat suitability area; blue, low habitat suitability area; yellow, moderate habitat suitability area; red, high habitat suitability area.

The potential distribution map of *P. viburni* was developed based on current speciation data and climate variables. Through the natural interval, the potential distribution range of *P. viburni* was divided into three levels (Figure 4). The highly suitable areas of *P. viburni*: in North America, it has a distribution in most parts of the southeastern coast and parts of the western coast of North America as well as scattered in other regions of the United States, Canada, and Mexico. In South America, it is concentrated in most of southern Brazil, all of Uruguay, central Chile, and the border of Brazil, Uruguay, Argentina, and Paraguay. It is scattered in other regions of South America. In the European continent, it is mainly distributed along the Mediterranean and Atlantic coasts. In mainland Asia, it is mainly distributed in provinces such as Hunan, Anhui, Jiangxi, Fujian, and Taiwan in China, as well as scattered along the coast of Japan and in areas near China, including Afghanistan, Pakistan, India, Nepal, Bangladesh, Bhutan, Myanmar, Laos, and Vietnam. Some coastal areas in Indonesia and near the Kuril Islands in Russia also exhibit its distribution. In the African continent, it is mainly distributed near the equator, and slightly distributed in southern South Africa, along the eastern coast of Madagascar, and in central Mozambique. In mainland Australia, the pest is mainly distributed in the southern region of Australia and spans most parts of New Zealand.

### 3.5. Future Potential Distribution

**Figure 5:**
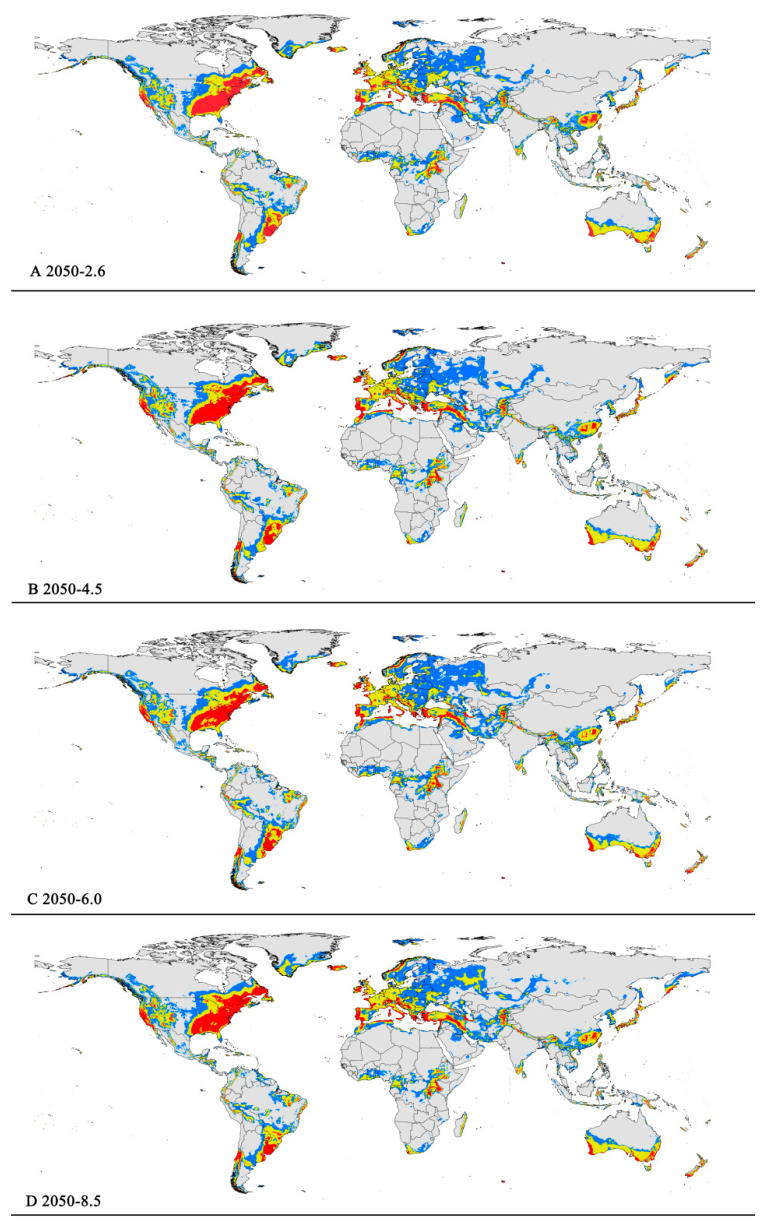
Future distribution models of *Pseudococcus viburni* on globe under different climate scenarios predicted in 2050 by MaxEnt. Gray, unsuitable habitat area; blue, low suitability area; yellow, moderate suitability area; red, highly suitability area. (**A**) RCP 2050−2.6; (**B**) RCP 2050-4.5; (**C**) RCP 2050-6.0; (**D**) RCP 2050-8.5.

**Figure 6:**
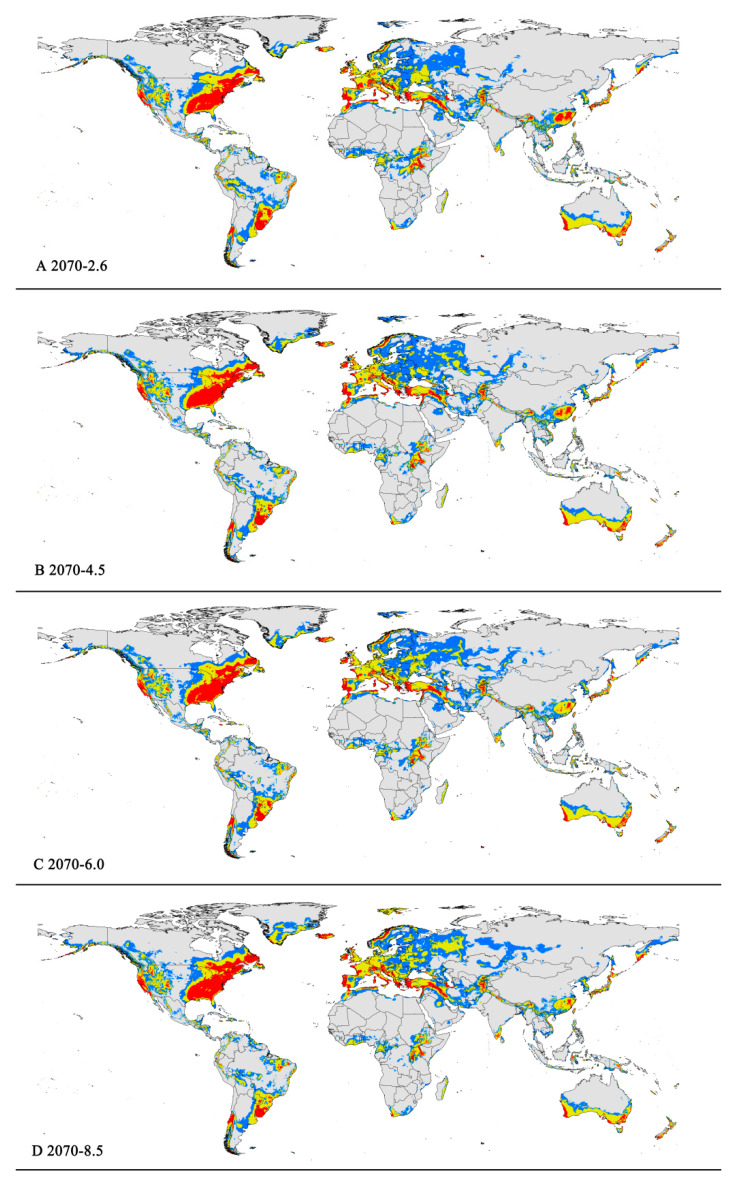
Future distribution models of *Pseudococcus viburni* on globe under different climate scenarios predicted in 2070 by MaxEnt. Gray, unsuitable habitat area; blue, low habitat suitability area; yellow, moderate habitat suitability area; red, highly habitat suitability area. (**A**) RCP 2070-2.6; (**B**) RCP 2070-4.5; (**C**) RCP 2070-6.0; (**D**) RCP 2070-8.5.

The potentially suitable areas for *P. viburni* under future scenarios (Figure 5 and Figure 6) were partially different from the current climatic conditions. In the future climate scenario, when compared with the current climate, the suitable areas showed different trends. From a global perspective, the highly suitable area showed a decrease trend under any of the future climate scenarios. When comparing the high suitability area values for all other climate scenarios with the current suitability area value, the decrease rate was more than 10%. In the RCP6.0-2050 and RCP6.0-2070 climatic scenarios, the area is −0.98 × 10^7^ km^2^ and −1.00 × 10^7^ km^2^, respectively (Figure 5C and Figure 6C and Table 3), and the decrease rate was 20.68% and 19.39%, respectively.

However, they all showed noticeable expansion in the low and moderate suitability areas. For moderately suitability areas, the prediction of MaxEnt about gain in the area under the future climate scenario combination RCP 8.5-2070 showed an increase of nearly 2.83 × 10^7^ km^2^ (a 12.27% expansion in the currently suitable habitat areas) (Figure 6D and Table 3). Except under the RCP4.5-2050 climate scenario, there was a slight decrease (3.50%), and under other climate scenarios, they all indicated a slight rise (1.17–5.19%) (Table 3). For low suitability area, under the RCP6.0-2050, RCP6.0-2070, and RCP8.5-2070 climate scenarios, the area increased to −3.29 × 10^7^ km^2^, −3.20 × 10^7^ km^2^, and −3.22 × 10^7^ km^2^, respectively, with growth rates of 24.75%, 21.55%, and 22.21%, respectively.

In summary, the total area of suitable habitat areas will tend to increase in the future. Under the RCP8.5-2070 climate scenario, the area of suitable habitat reached a maximum of 7.16 × 10^7^ km^2^, an increase of 11.84% compared to the current.

## 4. Discussion

Currently, there has been a wide use of modeling to predict species distribution in disciplines such as ecology, biogeography, weed science, and conservation biology [36]. Particularly, regarding biological invasions, many researchers have been applying species distribution models to predict the potential distribution of invasive species subsequently using research results to strengthen the monitoring of relevant countries and regions [37]. Compared to other models, MaxEnt software has out-performed other software (GARP, random forests, and Mahalanois) due to the need for presence-only data, stable performance, and a simple user interface [38]. In the results of this study, the MaxEnt method predicted the potential distribution of *P. viburni* based on the occurrence data of *P. viburni* and environmental variables under current and future climatic conditions in the world. The results based on the pROC test showed that the model had high performance—the prediction results of the model were much better than those of the random model.

The response curve in this study also reflected the relationship between *P. viburni* and relevant environmental variables, including the precipitation of the coldest quarter (Bio19), precipitation seasonality (Bio15), mean temperature of the wettest quarter (Bio8), mean diurnal range (Bio2), and precipitation of the warmest quarter (Bio18). The total area of suitable habitat is likely to increase in the future under different climate scenarios. Hence, the geographical areas that are supposed to provide suitable habitats for the pest in future should be focused on the future monitoring of the pest.

The research on the biological aspects of *P. viburni* has been insufficient. Despite this, Abbasipour and Taghavi confirm that the population density of *P. viburni* fluctuated with the seasons. The results show that the population density increased rapidly to an early peak in April, followed by a decline and then a low but steady density for remainder of the season until there was another decline in November in Iran [39]. In addition, as *P. viburni* populations move into the warmest summer months (June to July), the proportion of populations found underground and under the stem of tea trees increases. These data suggest that the *P. viburni* are seeking protection from the heat [39]. Based on these data and phenomena, we conclude that this species is more likely to survive in areas with relatively mild temperatures, and that either too high or too low temperature will affect its population density or survival. Our study suggests that the 19–24 °C for Bio8 (mean temperature of the wettest quarter) is an important variable determining the model. This seems to be consistent with the result of Abbasipour and Taghavi in 2007. Unfortunately, it seems we do not have enough biological data to speculate or verify the results of our model. Therefore, we can only look forward to future work to further validate our results.

Under the current climatic conditions, the pest is mainly distributed in the Hunan, Anhui, Jiangxi, Fujian, and Taiwan provinces in China. Currently, only Guizhou in China has records [40]. In the wake of global change, there is a possibility that the *P. viburni* may be introduced to environments that are conducive to its growth and development. This introduction will have disastrous impacts by providing the pest with opportunities to colonize and spread due to the lack of natural enemies in the introduced range. Even though the highly suitable area is small, the moderately and marginally suitable areas are large, indicating that *P. viburni* still poses an invasion risk in China. The moderately and marginally suitable areas are mainly located in South China, the quarantine measures at ports in these areas should be strengthened to prevent the invasion of *P. viburni*.

Recently, the main components of climate change have been global warming, drought, increased CO_2_ levels in the atmosphere and increased frequency of extreme weather events (IPCC, 2014). The impact of climate change on insects is particularly important as it can directly or indirectly affect their populations and distribution [26]. Hence, there is a need to keep an eye on the dispersal dynamics of invasive species under climate change. In this context, this study predicted the potentially suitable areas of *P. viburni* under the future climate scenario and compared it with the suitable areas under the current climatic conditions. The results showed that the total suitable area will increase in the future climate conditions. Therefore, the risk of *P. viburni* invasion will increase under the future climate scenarios. This is required to strengthen the monitoring and quarantine measures at ports in the southern region of China to strictly prevent the invasion of *P. viburni.*

Based on the theory of maximum entropy, the MaxEnt model was implemented using only two variables, i.e., occurrence information and corresponding environmental variables, yet the prediction results of the model in this study were relatively good [41]. In other words, the accuracy of the model based on MaxEnt was mainly affected by two factors: (i) occurrence data and (ii) environmental variables [42]. According to previous studies, areas for which more distribution data are available tend to be overpredicted, while areas with fewer or no distribution records will be underpredicted. The current model is based only on some abiotic factors, which limits the accuracy of the potential distribution range of species under climate change. Other factors will also affect the results of the model, such as the background area (also known as “research area”), dispersal capacity [43], land use [44], and the host’s distribution range. Further, in similar studies, some important factors are often ignored and the data required by the model are simplified, resulting in errors in the predicted results, and there is a huge difference between prediction and reality. Since different researchers apply different sample numbers and parameters to the model, even predictions for the same species may yield diverse outcomes, representing varied species [45].

This study provides important information about the potential distribution of *P. viburni* under climate change conditions. Moreover, this work also provides an important theoretical basis for the monitoring and control of *P. viburni.* Further, when more data on the species distribution become available, niche models will better predict the potential impact of climate change on this species’ distribution.

## 5. Conclusions

This study used the distribution data of *P. viburni* and environmental factors to predict potentially suitable regions for the invasion of this species under different climate scenarios. The results suggested that precipitation of the coldest quarter (Bio19), precipitation seasonality (Bio15), and mean temperature of the wettest quarter (Bio8) were the most important environmental factors determining the distribution of *P. viburni*. Under the current climate conditions, its potential suitable areas are mainly located in southern China, the whole of Japan, the North American continent (especially the eastern part of the United States), the southwestern part of South America, the Mediterranean coast, most of Europe, the central part of Africa, i.e., south of the Sahara Desert, and most of the southern coast of Australia. The total area of suitable habitat is likely to show an increased trend under future climatic conditions.

## Figures and Tables

**Table 1:** Environmental variables used in this study.

Variable Abbreviation	Variables
Bio1	Annual mean temperature (°C)
Bio2	Mean diurnal range (°C)
Bio3	Diurnal temperature difference to annual temperature difference ratio
Bio4	Temperature seasonality (standard deviation × 100)
Bio5	Max temperature of the warmest month (°C)
Bio6	Min temperature of the coldest month (°C)
Bio7	Temperature annual range
Bio8	Mean temperature of the wettest quarter (°C)
Bio9	Mean temperature of the driest quarter (°C)
Bio10	Mean temperature of the warmest quarter (°C)
Bio11	Mean temperature of the coldest quarter (°C)
Bio12	Annual precipitation (mm)
Bio13	Precipitation of the wettest month (mm)
Bio14	Precipitation of the driest month (mm)
Bio15	Precipitation seasonality
Bio16	Precipitation of the wettest quarter (mm)
Bio17	Precipitation of the driest quarter (mm)
Bio18	Precipitation of the warmest quarter (mm)
Bio19	Precipitation of the coldest quarter (mm)

The environmental variables used in this study are all climatic factors.

**Table 2:** Relative contribution of each environmental variable from MaxEnt.

Variables	Percentage Contribution (%)
Precipitation of the coldest quarter (Bio19)	**70.7**
Precipitation seasonality (Bio15)	**12.6**
Mean temperature of wettest quarter (Bio8)	**10.8**
Mean diurnal range (Bio2)	3
Precipitation of the warmest quarter (Bio18)	2.9

**Table 3:** Predicted suitable areas for *Pseudococcus viburni* under current and future climatic conditions (km^2^).

Pest Species	Habitat Level	Current Climate Conditions	Future Climate Conditions							
			rcp26-2050	rcp26-2070	rcp45-2050	rcp45-2070	rcp60-2050	rcp60-2070	rcp85-2050	rcp85-2070
obscure mealybug	LSA	−2.63 × 10^7^	−3.04 × 10^7^	−2.95 × 10^7^	−3.04 × 10^7^	−3.16 × 10^7^	−3.29 × 10^7^	−3.20 × 10^7^	−3.14 × 10^7^	−3.22 × 10^7^
			(15.24%)	(12.04%)	(15.59%)	(19.78%)	(24.75%)	(21.55%)	(19.5%)	(22.21%)
	MSA	−2.52 × 10^7^	−2.55 × 10^7^	−2.61 × 10^7^	−2.43 × 10^7^	−2.64 × 10^7^	−2.55 × 10^7^	−2.65 × 10^7^	−2.58 × 10^7^	−2.83 × 10^7^
			(1.17%)	(3.43%)	(−3.50%)	(4.69%)	(1.26%)	(5.19%)	(2.53%)	(12.27%)
	HSA	−1.25 × 10^7^	−1.09 × 10^7^	−1.07 × 10^7^	−1.09 × 10^7^	−1.05 × 10^7^	−0.98 × 10^7^	−1.00 × 10^7^	−1.11 × 10^7^	−1.11 × 10^7^
			(−12.34%)	(−14.38%)	(−12.58%)	(−15.53%)	(−20.68%)	(−19.39%)	(−10.70%)	(−10.98%)
	TSA	−6.40 × 10^7^	−6.68 × 10^7^	−6.62 × 10^7^	−6.57 × 10^7^	−6.85 × 10^7^	−6.83 × 10^7^	−6.86 × 10^7^	−6.83 × 10^7^	−7.16 × 10^7^
			(4.33%)	(3.51%)	(2.59%)	(6.97%)	(6.66%)	(7.14%)	(6.75%)	(11.84%)

Note: LSA: low suitability area; MSA: moderate suitability area; HSA: high suitability area; TSA: total suitability area.

## Data Availability

The data presented in this study are openly available in this manuscript.

## Supplementary Materials

The following supporting information can be downloaded at: https://www.mdpi.com/article/10.3390/insects15030195/s1, Figure S1: Partial AUC Values and Graphics, null model (red distribution), distribution of expectations created via bootstrapping replacement of 50% of the total available points and 1000 resampling replicates (blue distribution); Table S1: Raw data for distribution sites for *P. viburni*; Table S2: Distribution sites for *P.viburni* used in model; Table S3: Pearson’s correlation coefficients matrix of *P. viburni;* Table S4: ENMeval results for *P. viburni* from SDMs.
