# Supplementary figures and images for: Global Potential Distribution of Invasive Species Pseudococcus viburni (Hemiptera: Pseudococcidae) under Climate Change
Source: Insects. 2024 Mar 14;15(3):195. doi: 10.3390/insects15030195 (PMC10971524; doi:10.3390/insects15030195)

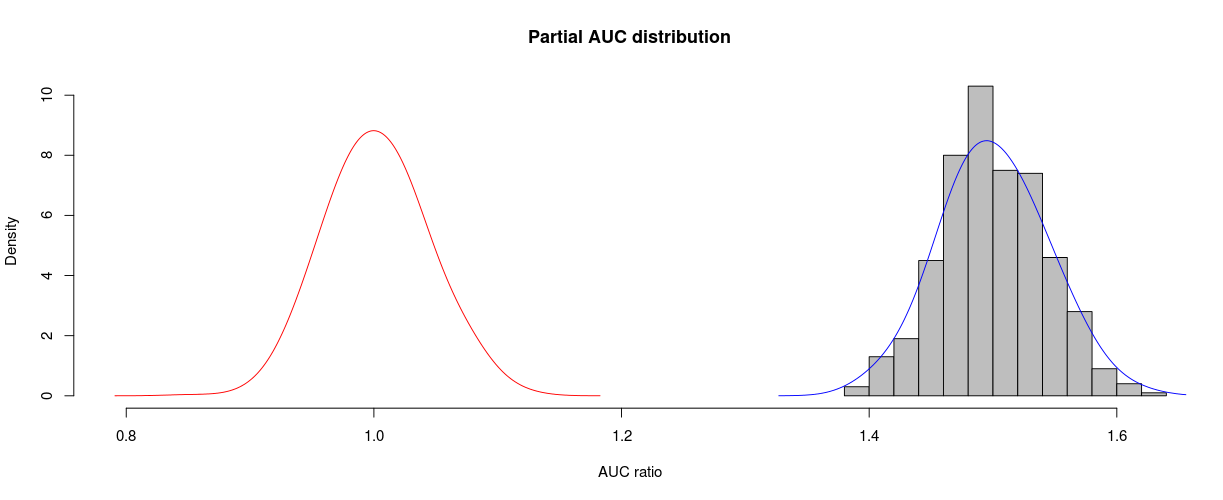

Supplement: Supplementary file 1 [file insects-15-00195-s001.zip › Supplementary File/Figure S1.png]
